# Fate of Viable but Non-culturable *Listeria monocytogenes* in Pig Manure Microcosms

**DOI:** 10.3389/fmicb.2016.00245

**Published:** 2016-03-02

**Authors:** Jérémy Desneux, Audrey Biscuit, Sylvie Picard, Anne-Marie Pourcher

**Affiliations:** ^1^Irstea-RennesRennes, France; ^2^Université Européenne de BretagneRennes, France

**Keywords:** *L. monocytogenes*, VBNC state, manure, microcosm, PMA, pyrosequencing

## Abstract

The fate of two strains of *Listeria monocytogenes* and their ability to become viable but non-culturable (VBNC) was investigated in microcosms containing piggery eﬄuents (two raw manures and two biologically treated manures) stored for 2 months at 8 and 20°C. Levels of *L. monocytogenes* were estimated using the culture method, qPCR, and propidium monoazide treatment combined with qPCR (qPCR_PMA_). The chemical composition and the microbial community structure of the manures were also analyzed. The strains showed similar decline rates and persisted up to 63 days. At day zero, the percentage of VBNC cells among viable cells was higher in raw manures (81.5–94.8%) than in treated manures (67.8–79.2%). The changes in their proportion over time depended on the temperature and on the type of eﬄuent: the biggest increase was observed in treated manures at 20°C and the smallest increase in raw manures at 8°C. The chemical parameters had no influence on the behavior of the strains, but decrease of the persistence of viable cells was associated with an increase in the microbial richness of the manures. This study demonstrated that storing manure altered the culturability of *L. monocytogenes*, which rapidly entered the VBNC state, and underlines the importance of including VBNC cells when estimating the persistence of the pathogens in farm eﬄuents.

## Introduction

The presence of *Listeria monocytogenes*, a food-borne pathogen, has been demonstrated in animal feces, manure and farm wastewaters (Farzan et al., 2010; Hellstrom et al., 2010; Boscher et al., 2012; Dungan et al., 2012; Pourcher et al., 2012). Humans can be exposed to *L. monocytogenes* strains by direct contact through farming operations or indirectly through consumption of food contaminated by manure from infected or shedding animals (Schlech et al., 1983; Nightingale et al., 2004; Mohammed et al., 2010). It has been reported that cattle farm ecosystems maintain a high prevalence of *L. monocytogenes*, including subtypes linked to human listeriosis (Nightingale et al., 2004). In a study conducted in piggeries in Brittany (France), Pourcher et al. (2012) observed high occurrence of *L. monocytogenes* in pig manures stored in pits and in lagoons after biological treatment (respectively, 18 and 24% of the samples were positive). Lyautey et al. (2007) showed a link between the occurrence of *L.* *monocytogenes* in surface waters in agricultural catchments in Ontario (Canada), the extent of cropped land and proximity to an upstream dairy operation. More recently, Stea et al. (2015), reported that surface waters may be a reservoir of *L. monocytogenes*, especially in rural agricultural watersheds. Farm environments may thus act as a vehicle for the dissemination of *L. monocytogenes*.

Several factors including temperature, moisture, pH and competition for nutrients may influence the survival of pathogenic bacteria (Guan and Holley, 2003). However, *L. monocytogenes* can acquire tolerance to numerous physical and physiochemical stresses and can thus survive in a wide range of environmental conditions (Roberts and Wiedmann, 2003; Gandhi and Chikindas, 2007), which may explain their high prevalence in stored and treated manures. Moreover, in response to environmental stress such as starvation, light, temperature, and NaCl concentrations, *L. monocytogenes* may become viable but non-culturable (VBNC; Besnard et al., 2002; Lindback et al., 2010). Although resuscitation from the VBNC state of *L. monocytogenes* is difficult to prove (Cappelier et al., 2007; Lindback et al., 2010), the presence of VBNC bacteria in the environment is a health risk for humans since the cells may retain their infectivity (Besnard et al., 2002).

In most studies on the survival of *L. monocytogenes* during manure storage, manure treatment (e.g., composting) or after manure is spread on soil, the viability of the pathogen was assessed only on the basis of its recovery by cultural methods (Nicholson et al., 2005; Grewal et al., 2007; Goberna et al., 2011; Erickson et al., 2014; Millner et al., 2014). Given the ability of *L. monocytogenes* to enter the VBNC state, the decrease in concentration reported by these authors may in fact underestimate the number of infectious pathogens. Very few studies have used molecular methods to quantify *L. monocytogenes* in urban or animal eﬄuents. Wery et al. (2006) and Klein et al. (2011) investigated the persistence of *L. monocytogenes* inoculated in microcosms in sewage sludge and in cattle manure (stockpiled or composted), respectively, using cultural methods and qPCR targeting *hlyA* gene. The results of these studies in which qPCR was used, showed a slower decline of *L. monocytogenes* cells than when a cultural method was used, suggesting the presence of non-culturable cells. However, DNA-based quantification methods detect DNA in both non-culturable and dead bacteria, leading to false positive results. To prevent the amplification of DNA from dead cells, propidium monoazide (PMA), a DNA-intercalating agent which can enter membrane-damaged cells, has been used in combination with qPCR (qPCR_PMA_) to enable only viable bacteria to be quantified (Nocker et al., 2006; Adela Yanez et al., 2011; Contreras et al., 2011; Dong et al., 2014; Li et al., 2014; Forghani et al., 2015). However, it is worth noting that qPCR_PMA_ remains difficult to apply in turbid matrices such as wastewater or manure containing high rates of suspended matter (Wagner et al., 2008; Varma et al., 2009). To overcome this drawback, our team has recently compared different experimental conditions to achieve optimal qPCR_PMA_ quantification of *L. monocytogenes* in pig manures (Desneux et al., 2015).

The aim of the present study was to evaluate the persistence of *L. monocytogenes* strains and their ability to enter the VBNC state during the storage of raw pig manure and biologically treated manure stored in a lagoon (lagoon eﬄuent) at the laboratory scale at two temperatures (8 and 20°C) selected to mimic the average winter and summer temperatures in piggery lagoons in Brittany. Manure and lagoon eﬄuent microcosms were inoculated with two rifampicin-resistant strains of *L. monocytogenes* originating from two piggeries, and quantified over a 63 days period by plate counts (cultivable cells), qPCR targeting *hlyA* gene (total cells) and qPCR after contact with PMA (viable cells). To better understand the relationship between the properties of the matrix and the behavior of *L. monocytogenes*, the chemical and microbial composition of the manures and the lagoon eﬄuents were analyzed at T0 and after 2 months of incubation.

## Materials and Methods

### Bacterial Strains

The experiments were carried out with two strains of *L. monocytogenes*: strain L111 (CIP 110869) and strain L120 (CIP 110870) isolated from pig manure and a lagoon eﬄuent, respectively. The two strains were serotyped by the research unit Hygiene and Quality of Poultry and Pork Products (Anses Ploufragan, France). They belonged to serogroups IVb (L111) and IIb (L120). Two rifampicin-resistant (Rifr) mutants (L111r and L120r) were generated from the two selected strains as described in Lung et al. (2001) to facilitate *L. monocytogenes* enumeration on plate agar. The Rifr strains were cultivated at 37°C in nutritive broth (OXOID, France) supplemented with 3 g L^-1^ of glucose and 0.1 g L^-1^ of rifampicin (Sigma–Aldrich, France; NBG-RIF broth). Both strains had similar growth rates in the nutritive broth.

### Characteristics of the Treatment Processes

Raw manures (Manures-1 and 2) and lagoon eﬄuents (Lagoons-1 and2) were collected in sterile flasks from pig manure treatment processing units at two farms (farms 1 and 2) located in Brittany (France). The two manure treatment consists of pre-storage of raw manure in a tank followed by centrifugation to remove phosphorus from the liquid phase. The recycled product (calcium or magnesium phosphate) is concentrated in a solid product mainly containing organic matter. The liquid phase is biologically treated in aerobic and anoxic stages in a reactor to reduce the level of nitrogen. The treated manure is dehydrated using a filter band press (farm 1) or settled in a settling tank (farm 2). The liquid fraction is then sent to an open-air lagoon where it is stored for 9–12 months before being used to water crops.

### Chemical Characterisation of Manure and Lagoon Eﬄuent

Total solids (TS), volatile solids (VS), total Kjeldahl nitrogen (TKN) and total ammonia nitrogen (TAN) were determined using standard methods (APHA, 1998). Volatile fatty acids (VFA) were analyzed using high-pressure liquid chromatography (HPLC; Peu et al., 2004). pH was measured using a hand-held pH meter (Hanna, Tanneries, France).

### Preparation of the Microcosms

Microcosm experiments were conducted in 200-mL glass bottles containing 100 mL of raw manure or lagoon eﬄuent. In order to simulate the storage condition of manure and lagoon, the bottles were closed with a screw plug to maintain anoxic conditions (raw manure microcosms) or with carded cotton plugs to allow air to penetrate (lagoon eﬄuent microcosms). The bottles were stored for a week in the dark at 8°C or 20°C to enable acclimation of endogenous microbial flora before inoculation with *L. monocytogenes* strains.

A volume of 150 μL of pure culture of the L111r or the L120r strain in the exponential phase growth in nutritive broth (Oxoid, France) was transferred into 100 mL NBG-RIF broth and incubated for 17 h at 37°C to obtain stationary-phase cells. Aliquots (40 mL) of the incubated NBG-RIF broth were centrifuged for 5 min at 5000 *g*. The pelleted cells were suspended in 5 mL of 0.8% NaCl and centrifuged for 5 min at 5000 *g*. The pellet was resuspended in 5 mL of 0.8% NaCl. Next, each microcosm was inoculated with 1 mL of the bacterial suspension to reach an initial concentration of approximately10^8^ *L. monocytogenes* mL^-1^. Three microcosms were used for each strain. A total of 24 microcosms (two strains, two raw manures, two lagoon eﬄuents, three replicates) were stored in the dark at 20 °C or 8°C for 63 days.

### Quantification of Autochthonous *L. monocytogenes* in Raw Manures and Lagoon Eﬄuents

*Listeria monocytogenes* was enumerated by a three-tube MPN method. Before inoculation with the Rifr strains, 10, 1, and 0.1 mL of raw manure or lagoon eﬄuent were transferred in 90 mL or 9 mL of ONE Broth-*Listeria* (Oxoid, France). After incubation for 48 h at 30°C, 0.1 ml of each enrichment broth was plated onto Rapid L’mono agar (Bio-Rad, France) and incubated for 48 h at 37°C. Characteristic colonies of *L. monocytogene*s were identified based on their phosphatidylinositol phospholipase C (PIPLC) activity.

### Quantification of Rifr Strains in the Microcosms

Samples for cultural, qPCR and qPCR_PMA_ quantification were collected in each microcosm immediately after inoculation of the Rifr strains (T0), and after 7, 21, 42, and 63 days of incubation.

#### Cultural Method

Serial 10-fold dilutions were prepared in peptone water (Oxoid, France). A volume of 0.25 mL of undiluted matrices or 0.1 mL of dilutions was surface plated on PALCAM agar without supplement (Oxoid, France) containing 100 mg L^-1^ of rifampicin and 50 mg L^-1^ of cycloheximide (Sigma–Aldrich, France). Colonies were counted after 48–72 h of incubation at 37°C. Results are expressed as cfu mL^-1^. The limit of detection was 4 cfu mL^-1^.

#### Propidium Monoazide (PMA) Treatment

Propidium monoazide (Biotium, Inc., Hayward, CA, USA) was dissolved in water to prepare a stock solution (1 mg mL^-1^) stored in the dark at -20°C. PMA was used as described in Desneux et al. (2015). Briefly, 400 μL of manure or 600 μL of lagoon eﬄuent were transferred in transparent polypropylene reaction tubes (Greiner Bio-One, France). PMA was added to the tubes containing manure or lagoon eﬄuent at a final concentration of 55 or 20 μM, respectively. After incubation in the dark at room temperature for 5 min (manure) or 20 min (lagoon eﬄuent), the samples were placed horizontally on ice at a distance of 20 cm from a 650-W halogen light source. They were exposed to the light source for 55 min (manure) or 30 min (lagoon eﬄuent). Then, 250 μL of PMA treated manure and 500 μL of PMA treated lagoon eﬄuent were transferred in 2 mL tubes and centrifuged for 5 min at 5000 *g*. The pellets were stored at -20°C before DNA extraction.

### DNA Extraction and Quantification of *L. monocytogenes* and Total Bacteria

DNA was extracted from PMA treated and untreated samples with Nucleospin kit for soil (Macherey Nagel) according to the manufacturer’s instructions with one modification of the elution procedure as described in Desneux and Pourcher (2014).

*Listeria monocytogenes* were quantified using the protocol of Nogva et al. (2000). Briefly, PCR reactions were performed in a volume of 25 μL containing 2 μL of diluted DNA, 1X TaqMan Buffer, 5 mM MgCl_2_, 200 μM dNTPs, 0.1 μM *L. monocytogenes*-specific probe, 0.3 μM *L. monocytogenes* specific primers (LiMo) (each), 12.5 μL of iQ Supermix (Bio-Rad, France) and water to complete the volume to 25 μL. Cycling parameters were 95°C for 10 min, followed by 40 cycles at 95°C for 20 s and 60°C for 60 s.

For total bacteria, PCR reactions were performed in a volume of 25 μL containing 12.5 μL of iQ SYBR Green Supermix (Bio-Rad, France), 200 nmol L^-1^ of each primer (W18 and W02), 2 μL of diluted DNA, and 9.5 μL of water. The cycling parameters were 95°C for 10 min, followed by 45 cycles at 95°C for 30 s, 60°C for 50 s, and 72°C for 30 s.

The primers and probes used are listed in **Table 1**. The PCR reaction was prepared using the Automated Pipetting System ep*Motion*^®^ (Eppendorf, France). PCR amplification was performed using a Bio-Rad CFX96 real-time PCR machine with Bio-Rad CFX Manager software, version 1.1 (Bio-Rad, France). Data were processed with Opticon Monitor version 3. 1. 32 and CFX manager version 1. 1 (Biorad, Hercule, CA, USA).

**Table 1:** Sequences of primers and probes used in the study.

Probe and primers	Sequences (5′–3′)	Tm (°C)	Reference
LiMo F	TGC-AAG-TCC-TAA-GAC-GCC-A	60.3	Nogva et al., 2000
LiMo R	CAC-TGC-ATC-TCC-GTG-GTA-TAC-TAA	60.3	Nogva et al., 2000
Probe *hlyA*	CGA-TTT-CAT-CCG-CGT-GTT-TCT-TTT-CG	70.2	Nogva et al., 2000
W18	GAGTTTGATCMTGGCTCAG	50	Godon et al., 1997
W02	GNTACCTTGTTACGACTT	50	Weisburg et al., 1991

Given that *hlyA* is present as a single copy in the genome of *L. monocytogenes* (Mengaud et al., 1988), the results are expressed as cfu equivalent (cfu-eq). Dilutions ranged from 4.5 × 10^8^ to 4.5 cfu-eq. Standard curves were generated by plotting threshold cycles (Ct) against cfu-eq. The detection threshold was 5.10^3^ cfu-eq mL^-1^ for manure and 2.5.10^3^ cfu-eq mL^-1^ for lagoon eﬄuent.

The cultural method and the qPCR performed on PMA treated samples and on untreated samples estimated:

– the culturable cells: plate counts
– the total number of cells: qPCR without PMA
– the number of viable cells: qPCR_PMA_
– the number of VBNC: [qPCR_PMA_] – [plate counts]

### T90 Calculations and Statistical Analysis

According to the curve characteristics, decay rates were estimated as T90 values using Chick’s model [monophasic model, *C*_(t)_ = *C*_0_ ×*e*^-kt^] or Cerf’s model [biphasic model, *C*_(t)_ = *C*_0_ × (*f* ×*e*^-k1t^ + (1-f) ×*e*^-k2t^] described in Xiong et al. (1999). *C*_(t)_ is the concentration at time t, *C*_0_ is the initial concentration, k is the first order decay rate constant, *f* is the initial proportion of the first fraction, k1 and k2 are the decay constants of the first and second phase, respectively. Decay rate models and their parameters were obtained using XLSTAT 2010 software. T90 (expressed in days) was calculated as follows: T90 = -ln (0.1)/k (Chick’s model) or T90 = -ln (0.1)/k1 (Cerf’s model). When the decay appeared after a lag, the T90 was calculated, taking into account the lag period. T90 were subjected to analysis of variance, and a Newman–Keuls test for multiple comparisons was applied to determine significantly different T90 (*p* < 0.05). Comparison of the Log_10_ reduction and T90 of the two strains was analyzed by means of the Student’s *t*-test (*p* < 0.01).

### Pyrosequencing of 16S rDNA Gene Sequences

The microbial diversity of the manure and the lagoon eﬄuent microcosms stored at 8 and 20°C was analyzed using a metagenomic approach applying 454-pyrosequencing technology. At T0 and T63, DNA extracted from the three replicate microcosms inoculated with strain L111r were pooled to obtain a total volume of 100 μL. DNA was precipitated with ethanol to reach a final volume of 20 μL. The 16S rDNA V3–V4 region of DNA was amplified with the primers F343 (5′-CTTTC CCTACACGACGCTCTTCCGATCTTACGGRAGGCAGCAG-3′) and R784 (5′-GGAGTTCAGACGTGTGCTCTTCCGATCTT ACCAGGGTATCTAATCCT-3′) using 30 amplification cycles with an annealing temperature of 65°C. The average amplicon length was 510 bp. The 16S rDNA V3–V4 region of the DNA extracts was sequenced on the GeT-PlaGe platform in Toulouse (Genotoul, Toulouse, France) using Illumina Miseq technology. Because MiSeq enables paired 250-bp reads, the ends of each read overlap and can be stitched together to generate extremely high quality, full-length reads of the entire V3 and V4 region in a single run. Single multiplexing was performed using a homemade 6 bp index, which was added to the R784 during a second PCR with 12 cycles using forward primer (AATGATACGGCGACCACCGAGATCTACACTCTT TCCCTACACGAC) and reverse primer (CAAGCAGAAGA CGGCATACGAGAT-index-GTGACTGGAGTTCAGACGTGT). The resulting PCR products were purified and loaded onto the Illumina MiSeq cartridge according to the manufacturer’s instructions. The quality of the run was checked internally using PhiX, and then each paired-end sequence was assigned to its sample with the help of the previously integrated index.

### Analysis of Pyrosequencing Data Using the QIIME Pipeline

The data were studied using the quantitative insights into microbial ecology (QIIME) pipeline (Caporaso et al., 2010b). Operational taxonomic units (OTUs) were formed at 97% similarity using UCLUST (Edgar, 2010). The representative sequences of each OTU were aligned to 16S reference sequences using PyNAST with a minimum length of 150 bp and 75% minimum percent identity (Caporaso et al., 2010a). The alpha diversity within the microcosm samples was estimated by the Chao1 richness estimator, the abundance-based coverage (ACE) estimator and the Shannon index. Shifts in bacterial community structure over time were assessed by principal coordinate analysis (PCoA) of the pairwise weighted Unique Fraction (UniFrac) distances (Lozupone and Knight, 2005; Lozupone et al., 2006, 2011). Uncertainty in PCoA plots was estimated using jackknife analysis, in which the jackknife replicate was 10.

## Results

Autochthonous *L. monocytogenes* were not detected (<4 MPN 100 mL^-1^) in the four matrices collected for the microcosm assays.

### Persistence of Inoculated Strains in Manures and Lagoons

**FIGURE 1:**
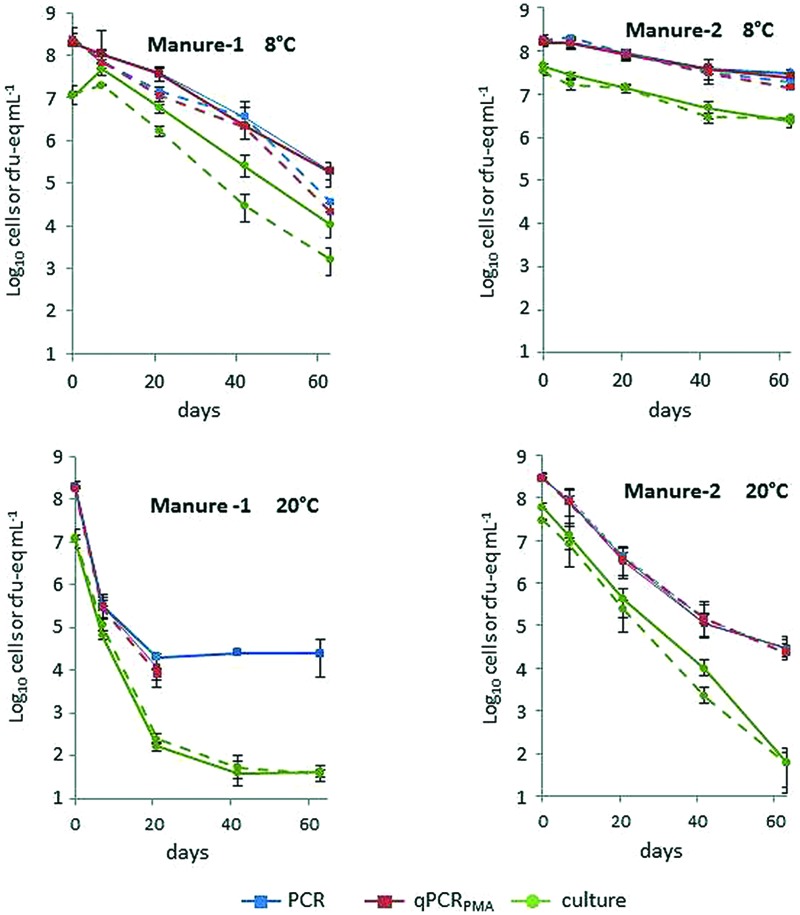
**Average concentrations of strains L111r (solid line) and L120r (dotted line) in Manure-1 and Manure-2 incubated at 8 and 20°C by qPCR, qPCR_PMA_ and culture.** The bars represent minimum and maximum values of triplicates.

**Figure 1** shows the levels of strains L111r and L120r for each culture and molecular method in the two manures stored at 8 and 20°C. A decrease in the concentration of *L. monocytogenes* was observed in all microcosms but both strains were still recovered 63 days after the inoculation. The two strains displayed similar behavior depending on the temperature and on the origin of the manure (**Figure 1**, Supplementary Table S1). Generally, the lowest temperature increased the persistence of both strains, regardless of the quantification method used. The Log_10_ reduction observed between 8 and 20°C was more marked using plate counts. The biggest difference was observed in Manure-2 microcosms in which the average reduction in culturable bacteria did not exceed 1.2 Log_10_ at 8°C whereas it reached 6.2 Log_10_ after 2 months of incubation at 20°C (**Table 2**). T90 values observed with both cultural and molecular methods were approximately 2.5-fold lower in Manure-1 than in Manure-2. Except for cultivable cells at 20°C, the Log_10_ reduction after 63 days was also higher in Manure-1 (3.4–3.8 at 8°C; ≥4.4 at 20°C) than in Manure-2 (0.9–1.2 at 8°C; 4.0 at 20°C). Moreover, the decrease in the concentration of both strains at 20°C showed a marked biphasic pattern in Manure-1, which was not observed in Manure-2, with a break in the kinetics after 21 days of incubation.

**Table 2:** T90 values (days) and Log_10_ reduction after 63 days for the two strains of *Listeria monocytogenes* (mean of 6 values) in manure and lagoon eﬄuent microcosms maintained at 8 and 20°C.

Matrix	Method	T90 (days)				Log_10_ reduction			
		8°C		20°C		8°C		20°C	
		Mean	*(SD)*	Mean	*(SD)*	Mean	*(SD)*	Mean	*(SD)*
Manure-1	Culture	21.2^e^	(1.7)	3.6^b^	(0.6)	3.5^abc^	(0.6)	5.5^b^	(0.2)
	qPCR	19.3^ef^	(3.6)	2.5^b^	(0.2)	3.4^abc^	(0.4)	4.4^c^	(0.5)
	qPCR	18.9^ef^	(5.9)	2.5^b^	(0.2)	3.8^a^	(0.7)	4.6	
Manure-2	Culture	53.5^a^	(4.9)	9.9^a^	(1.5)	1.2^fg^	(0.2)	6.2^a^	(0.5)
	qPCR	56.7^a*^	(2.1)	12.1^a^	(1.1)	0.9^g^	(0.1)	4.0^c^	(0.2)
	qPCR	51.7^a*^	(5.7)	11.2^a^	(1.1)	1.1^fg^	(0.1)	4.0^c^	(0.2)
Lagoon-1	Culture	18.4^ef^	(3.6)	10.3^a^	(2.8)	3.6^ab^	(0.6)	4.0^c^	(0.7)
	qPCR	17.1^ef^	(2.5)	10.7^a^	(2.9)	2.9^c^	(0.2)	2.5^d^	(0.2)
	qPCR	15.2^f^	(3.3)	10.0^a^	(3.3)	3.2^bc^	(0.2)	2.8^d^	(0.2)
Lagoon-2	Culture	25.7^d^	(0.5)	10.6^a^	(1.0)	2.4^d^	(0.3)	5.9^a^	(0.4)
	qPCR	37.1^b^	(2.8)	12.2^a^	(1.8)	1.6^ef^	(0.2)	4.3^c^	(0.2)
	qPCR	32.4^c^	(1.4)	10.1^a^	(1.5)	2.0^de^	(0.2)	4.4^c^	(0.3)

Values in the same column followed by different letters differ significantly (Newman–Keuls. *p* < 0.05); *mean of 3 values (strain L120r); † detected but not quantified.

**FIGURE 2:**
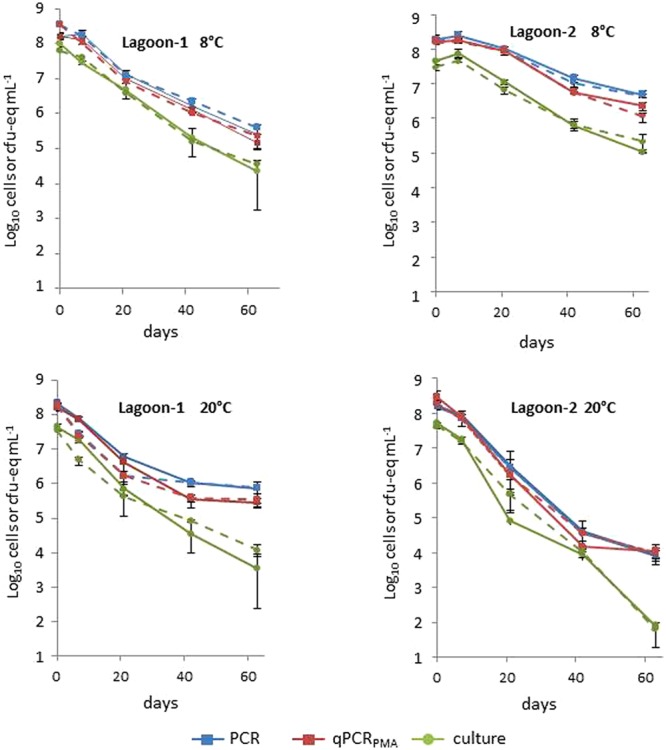
**Average concentrations of strains L111r (solid line) and L120r (dotted line) in Lagoon-1 and Lagoon-2 incubated at 8 and 20°C by qPCR, qPCR_PMA_ and culture.** The bars represent minimum and maximum values of triplicates.

The two strains also exhibited similar behavior in the lagoon microcosms regardless of the method of quantification (**Figure 2**, Supplementary Table S1). The T90 values and the Log_10_ reduction were affected by the origin and the temperature of the lagoon, but to a lesser extent than that observed in manure microcosm assays. At 8°C, both strains persisted better in Lagoon-1 than in Lagoon-2 whereas the opposite was observed at 20°C. Both strains persisted longer at 8°C in Lagoon-2 with a T90 of between 25.7 and 37.1 days at 8°C, and between 10.1 and 12.2 days at 20°C. The Log_10_ reduction was also lower at 8°C (1.6–2.4) than at 20°C (4.3–5.9) after 2 months of incubation. In contrast, the persistence of the strains in Lagoon-1 was hardly influenced by the temperature, leading to a similar Log_10_ reduction (approximately 3.1) at the end of the incubation period.

### Comparison of the Level of the Cultivable, Viable, and Total Bacteria

Considering the strains together, there was no significant difference between the T90 values obtained with the cultural and molecular methods at 20°C (*p* > 0.05), regardless of the matrix, whereas the Log_10_ reduction was systematically significantly higher (*p* < 0.05) after 63 days when estimated using cultural method (**Table 2**). At 8°C, the same trend was observed, but the differences between the Log_10_ reductions were less marked than at 20°C.

At T0, the difference in *L. monocytogenes* concentrations measured with the cultural method and with qPCR_PMA_ ranged from 0.5 to 0.7 Log_10_ in the lagoon eﬄuent microcosms and from 0.7 to 1.3 Log_10_ in the manure microcosms. Interestingly, the biggest differences in concentration obtained between culture and qPCR_PMA_ were observed in Manure-1. Except for Manure-1 at 8°C, for which growth of cultivable bacteria was observed during the first week of incubation, cultivable, total and viable cells exhibited similar behavior during the 21 first days. After 3 weeks, in all microcosms at 20°C and in two microcosms at 8°C, the levels of cultivable cells declined faster than viable bacteria, pointing to an increase in VBNC forms over time.

Considering the viable cells, the proportion of VBNC cells at T0 varied between the strains but was significantly higher (*p* < 0.05) in manures (81.5–94.8%) than in lagoon eﬄuents (67.8–79.2%; **Table 3**). Their proportion changed over time. The biggest difference in the proportion of VBNC between T0 and T63 was observed in lagoon eﬄuents at 20°C (18–21%) and the smallest in raw manures at 8°C (0.5–3.5%), suggesting that *L. monocytogenes* kinetics of entry into the VBNC state depended on the matrix and on the temperature.

**Table 3:** Percentage of VBNC cells among viable cells in the two strains of *L. monocytogenes* (mean of 6 values) at 8 and at 20°C in manure and lagoon eﬄuent microcosms at T0 and T63 days.

Temperature (°C)	Matrix	T0		T63	
		Mean	*(SD)*	Mean	*(SD)*
8	Manure-1	94.5^a^	(1.9)	95.0^a^	(2.1)
8	Manure-2	81.5^b^	(5.9)	85.0^b^	(6.3)
20	Manure-1	94.8^a^	(2.2)	98.8^a^	(0.9)
20	Manure-2	84.8^ab^	(5.9)	99.8^a^	(0.2)
8	Lagoon-1	67.8^c^	(17.2)	83.1^b^	(2.6)
8	Lagoon-2	76.1^bc^	(5.4)	88.7^b^	(8.3)
20	Lagoon-1	79.2^bc^	(4.6)	97.3^a^	(3.0)
20	Lagoon-2	78.1^bc^	(5.0)	99.2^a^	(0.7)

*Values in the same column followed by different letters differ significantly (Newman–Keuls. p < 0.05).*

In all the microcosms, quantification by qPCR gave similar results as by qPCR_PMA_, indicating that the bacteria remained viable in both manure and lagoon eﬄuent microcosms. The concomitant decline in total and viable bacteria suggests that the DNA of dead bacteria was rapidly degraded or bound to the matrix, thereby preventing its amplification. To test this hypothesis, a complementary experiment was performed. A culture of *L. monocytogenes* washed with sodium chloride and exposed to ultrasound (8 min, 360 W) was placed in a water bath for 10 min at 85°C to damage the bacterial membrane. Manure and lagoon eﬄuent were inoculated in triplicate with dead bacteria at an initial concentration of 7 107 cfu mL-1. The manure and lagoon eﬄuent were then incubated at 20°C. The cell numbers measured by qPCR dropped by more than 3.2 Log_10_ within 5 days, confirming the rapid degradation of the DNA of dead bacteria in both the manure and lagoon eﬄuent.

The conditions of each microcosm led to a particular behavior of the culturable and VBNC cells, suggesting that the persistence of *L. monocytogenes* was affected by the combined influence of the temperature and of the composition of the matrix.

### Chemical and Microbial Composition of the Manures and Lagoon Eﬄuents

The chemical composition of the four matrices differed but remained stable over the course of the experiment (Supplementary Table S2) and was not affected by the incubation temperature. The pH ranged between 7.3 and 7.8 in the manures and between 8.0 and 9.0 in the lagoon eﬄuent. Organic matter and nitrogen contents measured in manures ranged between 5.4 and 18.6 g kg-1 and between 1.8 and 4 gN kg-1, respectively, and were 10-fold higher than in lagoon eﬄuents. The level of total bacteria estimated by qPCR_PMA_ was also 10-fold higher in the manures than in lagoon eﬄuents. At T0, they ranged from 9.3 to 9.8 Log_10_ cfu-eq mL-1 in the manures and from 8.4 to 8.7 Log_10_ cfu-eq mL-1 in the lagoon eﬄuents and did not change throughout the incubation period.

Since the behavior of the two strains was similar, the bacterial community profiles at T0 and T63 were assessed by 16S rRNA pyrosequencing of the microcosms inoculated with strain L111r. The observed OTU numbers and richness estimators (Chao 1 and ACE), showed that the richness values varied four and sevenfold among the microcosms, respectively (**Table 4**). The richness of the manure (4428–5470 observed OTUs) and the species diversity estimated by the Shannon index (8.2–9.3) decreased slightly during incubation. The richness of the lagoon eﬄuent microcosms was more variable, ranging from 1388 to 4540 OTUs. The lowest diversity was observed in Lagoon-1 at 8°C, which also displayed the lowest richness. In contrast to the manure, the diversity of Lagoon-2 increased at 8°C in the microcosms, or remained stable throughout incubation.

**Table 4:** Operational taxonomic units (OTU), Chao 1 (species richness estimator), ACE (abundance-based coverage estimator), and Shannon (diversity index) of samples calculated at T0 and after 63 days of incubation for each microcosm inoculated with strain L111r.

Matrix	Temperature (°C)	Time (days)	Observed OTUs	Chao1	ACE	Shannon
Manure-1	8	0	5470	19965	23270	9.5
	8	63	5171	18536	21308	8.2
	20	0	5280	12371	15136	8.3
	20	63	4750	13233	15987	9.0
Manure-2	8	0	4872	15451	17821	8.9
	8	63	4428	14046	16234	8.5
	20	0	4962	15625	17912	9.0
	20	63	4434	14431	16490	8.6
Lagoon-1	8	0	1388	3090	3329	4.3
	8	63	2007	5231	5272	8.0
	20	0	2659	6945	7842	6.5
	20	63	2975	7780	8810	7.4
Lagoon-2	8	0	4540	14744	16621	8.5
	8	63	4157	13731	15194	8.5
	20	0	1989	5198	5505	6.6
	20	63	3676	10293	11608	8.3

**FIGURE 3:**
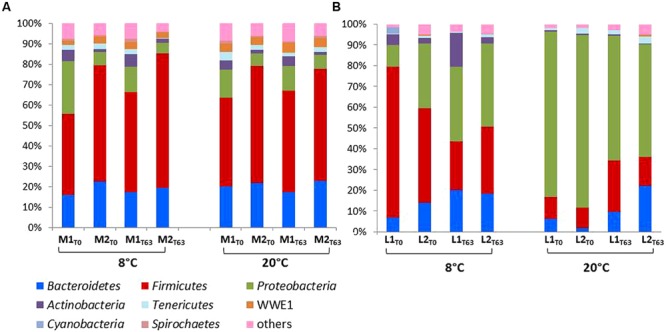
**Relative abundances of major phyla in manures **(A)** and lagoon eﬄuents **(B)** microcosms at days T0 and T63.** Only relative abundances of phylum higher than 1% are shown, all other sequences are included in “others.”

**FIGURE 4:**
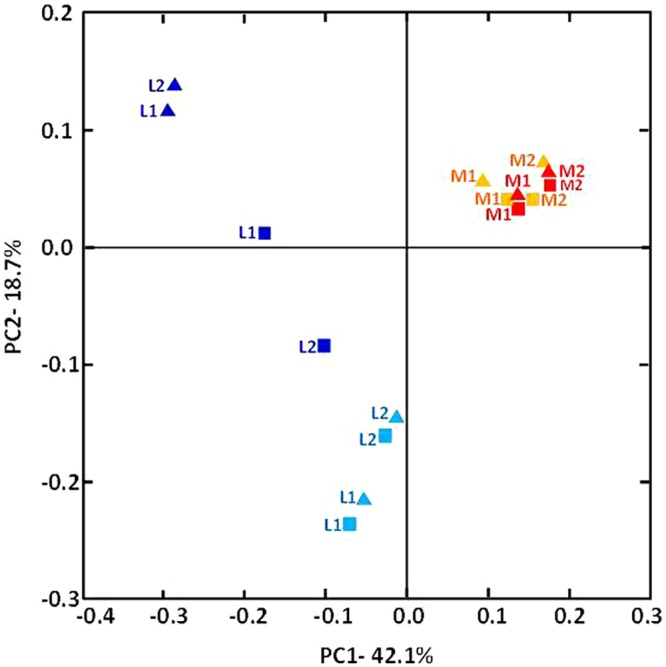
**β-diversity analysis of the composition of the manures and lagoon eﬄuents inoculated with strain L111r.** The phylogenetic dataset was analyzed using Jackknifed PCoA of the weighted pairwise UniFrac distance. Manure-1 (M1) and Manure-2 (M2) at 8°C (orange), M1 and M2 at 20°C (red), lagoon-1 (L1), and lagoon-2 (L2) at 8°C (pale blue), L1 and L2 at 20°C (dark blue); triangles represent T0; squares represent T63.

**FIGURE 5:**
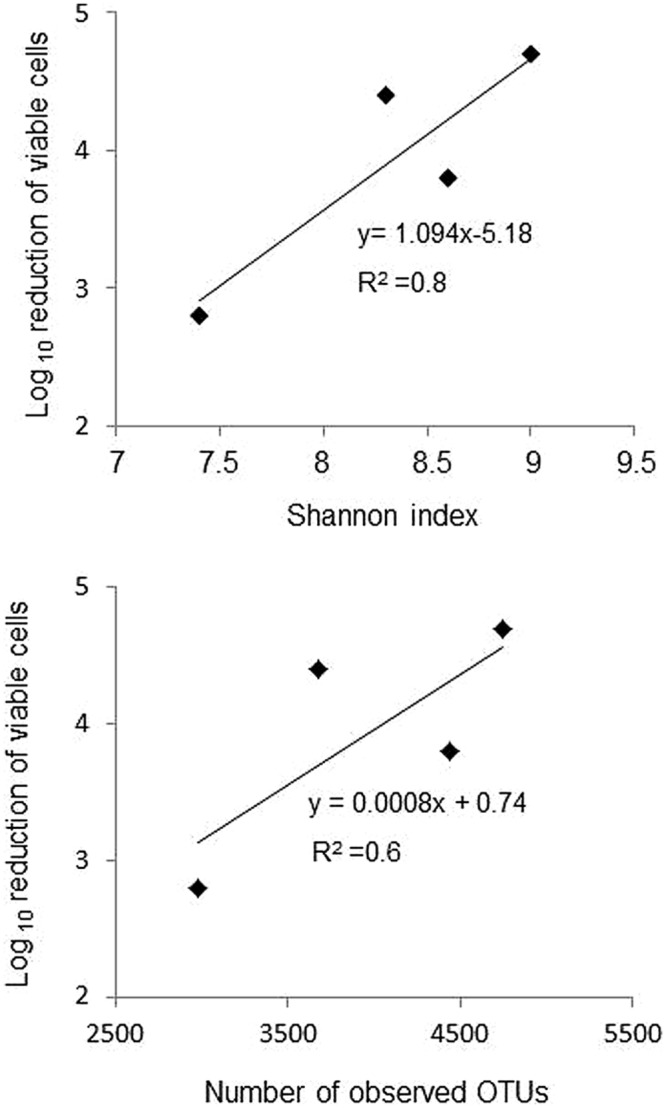
**Relationship between Log_10_ reduction of viable cells of strain L111r estimated by qPCR_PMA_ incubated at 20°C and the Shannon index and the numbers of observed OTUs at T63**.

The relative taxonomic abundance of the bacterial community at the phylum level is presented in **Figure 3** and Supplementary Table S3. Samples were dominated by four major phyla found in both manure and lagoon microcosms: *Proteobacteria* (7–26% in manures; 10.5–83% in lagoons), *Firmicutes* (39–66% in manures; 10–72.5% in lagoons), *Bacteroidetes* (16–23% in manures; 1.8–22% in lagoons) and *Actinobacteria* (1.4–6.4% in manures; 0.4–16% in lagoons). The relative abundance of the dominant phyla was more stable in manures than in lagoon eﬄuents which varied between the two incubation temperatures. PCoA of the unweighted Unifrac distance (**Figure 4**) revealed significant differences between the β diversity of the manure and lagoon microcosms. The manures clustered close together, showing that their bacterial communities remained stable over time regardless of temperature, whereas the lagoon eﬄuents were more separated. At 8°C, the overall composition of the microbiota in both lagoons was relatively stable, whereas changes in the communities occurred at 20°C. Given the stability of the bacterial communities in the manures, it appears difficult to establish a link between the behavior of the strains and the relative taxonomic abundances of the bacterial community of the matrices. However, the Log_10_ reduction in the number of viable cells of *L. monocytogenes* strain L111r estimated by qPCR_PMA_ increased with an increase in species diversity and in the number of observed OTUs (**Figure 5**).

## Discussion

Little information is available on the survival and the ability of *L. monocytogenes* to enter in the VBNC state during storage of the manure. In this study, the persistence of two strains of *L. monocytogenes* originating from piggery eﬄuents was studied in raw manure and in lagoon eﬄuent (liquid fraction of biologically treated manure) to see if storing the eﬄuents favors the formation of VBNC cells. Both strains were still detected after 63 days of incubation regardless of the conditions of the microcosm, indicating a high potential for the persistence of *L. monocytogenes* in manures and their liquid fraction after biological treatment. The inactivation rates did not depend on the origin of the strain (manure or lagoon eﬄuent) or of their serogroup (IIb, IVb). A similar trend has been observed in digested sludge inoculated at 35°C with other pathogens. Three strains of *Salmonella* and three strains of *Escherichia coli* underwent similar decay over an incubation period lasting 20 days, irrespective of the origin of the strain (Smith et al., 2005). Similarly, Garrec et al. (2005) found no difference in survival between two strains of *L. monocytogenes* inoculated in sewage sludge stored at 20°C for 70 days. However, McLaughlin et al. (2011) observed different behaviors of three strains of *L. monocytogenes* belonging to three serogroups inoculated in a soil stored at 25°C or at 30°C for 6 days, suggesting that, depending on the type of matrix inoculated, the intrinsic characteristics of the strains may have an effect. It is known that the persistence of pathogenic bacteria in the environment depends on biotic and abiotic parameters and on their interaction. In our study, the properties of the matrices appeared to mainly impact the survival of the two strains.

### Effect of the Matrix

Although the lagoon eﬄuent microcosms allowed air to penetrate and the concentrations of VS and nitrogen in these matrices were 10 times lower than in the raw manures, no clear difference in the survival rate was observed between these two types of eﬄuents. At 8°C, the survival of culturable and viable *L. monocytogenes* differed with the origin of the eﬄuent (farm 1 or farm 2) rather than between raw and treated manures. The T90 values were lower in the eﬄuents collected from farm 1 than in the eﬄuents from the farm 2. At 20°C, except for the T90 value observed in Manure-1, which was significantly lower than that observed in the three other matrices, the differences in the behavior of the strains between the eﬄuents from the two farms were less marked. Since the chemical and microbial parameters of the manures were similar, there is no apparent explanation for the particular biphasic decay of cultivable *L. monocytogenes* observed in Manure-1 at 20°C.

The effect of the matrix on the survival of *L. monocytogenes* has also been investigated in different types of matrices. Lemunier et al. (2005) and Paniel et al. (2010) observed that the persistence of *L. monocytogenes* in composts depended on the degree of maturation of the inoculated matrices. Likewise, Klein et al. (2011) reported T90 values for *L. monocytogenes* (determined using the cultural method) of 11 days in stockpiled manure and 17 days in composted manure maintained at 20°C. Our results highlight the combined effect of the matrix and the temperature on the survival of *L. monocytogenes*, which has also been observed in soil and in manured soil inoculated with a strain of *Salmonella* Typhimurium (Garcia et al., 2010). In that case, the presence of manure in the soil significantly reduced the survival of the strain of *Salmonella* at 5°C and 15°C but not at 25°C. Furthermore, characteristics of manure matrix can also contribute to the survival of pathogens as suggested by Diao et al. (2015) who observed that temperature, moisture content and particle size of dairy compost affected the survival of *E. coli* O157:H7 and *Salmonella* Typhimurium.

### Effect of Temperature

As expected, the lowest temperature increased the survival of *L. monocytogenes*. The effect of temperature on the persistence of pathogenic or enteric bacteria is well known and has been reported for *Salmonella, Campylobacter coli, L. monocytogenes*, and *E.coli* in livestock waste, in manure, and in manured soil (Mawdsley et al., 1995; Himathongkham et al., 1999; Arrus et al., 2006; Semenov et al., 2007; Garcia et al., 2010; Bui et al., 2011; Klein et al., 2011). In bovine manure-amended soil, Jiang et al. (2004) detected *L. monocytogenes* up to 43 days at 5°C, and up to 21 days 21°C. However, in our study, the impact of the temperature varied with the matrix. Indeed, the difference in Log_10_ reduction for a given matrix after 63 days of incubation at between 8 and 20°C ranged from 0.4 Log_10_ (Lagoon-1) to 5 Log_10_ (Manure-2). This is consistent with the results reported by Klein et al. (2011), who compared the behavior of an inoculated strain of *L. monocytogenes* in microcosms containing stockpiled or composted manure. After 10 days of incubation, the Log_10_ reduction in composted manure decreased by 4 at 37°C and by 1.5 at 20°C, and in stockpiled manure, it decreased by 3.5 at 37°C and by 2 at 20°C.

### Effect of Autochthonous Flora

After the manures and lagoon eﬄuents were acclimatized at the two temperatures for 8 days, the composition of the autochthonous flora in the manures was similar at 8 and 20°C, but differed in the lagoon eﬄuents. The three major OTUs (*Firmicutes, Bacteroidetes*, and *Proteobacteria*) found in the two farm eﬄuents were also dominant in the surface crust of swine slurry (Duan et al., 2014). Lu et al. (2014) also reported a high proportion of *Firmicutes* and *Bacteroidetes* in pig and piglet manures, whereas in their study *Proteobacteria* accounted for less than 1.5% of identified taxa. At T0, *Firmicutes* dominated in manures at both temperatures and in lagoon eﬄuents at 8°C. This phylum was less abundant in lagoon eﬄuents at 20°C in which *Proteobacteria* dominated. After 2 months of incubation, the relative abundance of the dominant phyla in the manures and the lagoon eﬄuents remained relatively stable. The manures showed higher diversity than the lagoon eﬄuents, indicating that the biological treatment and the subsequent storage of the liquid eﬄuent reduced both richness and diversity. The behavior of *L. monocytogenes* (especially the low T90 value observed in Manure-1 at 20°C) does not appear to be influenced by the taxonomic composition of the manures and lagoon eﬄuent communities. Nevertheless, interestingly, the decline in the number of viable cells of strain L111r increased with an increase in species diversity and in the number of OTUs. This finding is in agreement with the results of a study by Vivant et al. (2013), who reported that the survival of *L. monocytogenes* in soil microcosms decreased with the diversity and abundance of bacterial communities.

### Formation of VBNC in Manure and Lagoon Eﬄuent

The persistence of *Listeria* in environmental matrices (soil, whether manured or not, compost, manure) is mainly estimated by culture methods (Hutchison et al., 2005; Lemunier et al., 2005; Nicholson et al., 2005; Paniel et al., 2010; Piveteau et al., 2011; Locatelli et al., 2013; Vivant et al., 2013). Yet several studies on food products, biofilms, and water, showed that *L. monocytogenes* can enter the VBNC state (Besnard et al., 2000, 2002). Moreover, the presence of a VBNC indicator or of pathogenic bacteria in sludge has been suggested by comparing the results of culture and qPCR methods (Higgins et al., 2007; Viau and Peccia, 2009; Erkan and Sanin, 2013). But only a few studies have demonstrated the presence of indicator or pathogenic bacteria in the VBNC state in sludge or in manured soil using PMA or RNA based techniques (Jiang et al., 2013; Fu et al., 2014). Although the use of qPCR_PMA_ to detect viable bacteria in sludge is limited by problems with turbid matrices (Wagner et al., 2008), we previously demonstrated that qPCR_PMA_ can be applied to manures after optimisation of the conditions of exposure (Desneux et al., 2015). In the present study, analysis of cell viability in the microcosms, conducted using PMA, showed that *L. monocytogenes* entered the VBNC state in piggery eﬄuents. The number of VBNC cells in broth before inoculation was not estimated. However, although the strains were inoculated in similar condition in all experiments (early stationary-phase cells), different proportions of VBNC cells among viable cells were observed at T0 (ranged from 68 to 79% in lagoons and from 81.5 to 95% in manures). These results suggested that the loss of culturability depended on the type of matrix in the first hour of contact. The rapid appearance of VBNC cells we observed in the microcosms is in agreement with the work of Piveteau et al. (2011), who used whole-genome microarrays to analyze transcriptome modifications in a soil extract inoculated with a strain of *L. monocytogenes*. Their analysis revealed massive transcriptional modifications within 30 min of incubation. In our study, although viable bacteria decreased over time in all the microcosms, the proportion of VBNC increased. The kinetic of appearance of VBNC cells depended on the matrices. Manures appeared to be more favorable to a switch in *L. monocytogenes* cells to VBNC than lagoon eﬄuents within the first hours of contact whereas after 63 days of incubation, the proportion of VBNC was similar in the two types of matrix. Furthermore, the proportion of VBNC was higher at 20°C than at 8°C. This may reflect the greater ability of this psychrophilic bacterium to survive and to maintain culturability at low temperatures. It is noteworthy that culturability also depends on the physiological state of the inoculated bacteria. Thus, we observed that when *L. monocytogenes* cells in the exponential phase were used as the inoculum for the microcosms instead of stationary cells, the proportion of VBNC at T0 decreased by 14% (data not shown).

The comparison of quantification of viable and total *L. monocytogenes* cells by qPCR_PMA_ and qPCR clearly showed that extracellular DNA was rapidly degraded in these environments, providing an additional source of nutrients. The rapid disappearance of DNA we observed at 20°C is supported by data in the literature, which also showed a high recycling rate of extracellular DNA in manure (Lebuhn et al., 2004; Klein et al., 2011).

## Conclusion

This study showed that the survival of *L. monocytogenes*, which was shorter at 20°C than at 8°C, did not depend on the serotype or on the origin of the strain. Although lagoon eﬄuents were less concentrated than manures, this did not impair the survival of *L. monocytogenes*. Here, for the first time, we demonstrate the ability of *L. monocytogenes* to become VBNC in manures and to persist in this state for at least 2 months. The high proportion of VBNC, which increased over time and reached 99.8% of viable bacteria, confirmed that the culture method currently used to detect pathogens in manured material results in underestimation of the survival rate of pathogens during storage of manure. The PMA based method appears as a promising approach to assess the potential health risk associated with manure handling.

## Author Contributions

JD and A-MP designed laboratory work. JD performed microbiology laboratory work, as well as drafting the manuscript. A-MP finalized the manuscript. SP performed the chemical analyses. AB participated to a part of the microbiology laboratory work. All authors read and approved the final manuscript.

## Conflict of Interest Statement

The authors declare that the research was conducted in the absence of any commercial or financial relationships that could be construed as a potential conflict of interest.
