# Supplementary material for: Fate of Viable but Non-culturable Listeria monocytogenes in Pig Manure Microcosms
Source: Front Microbiol. 2016 Mar 2;7:245. doi: 10.3389/fmicb.2016.00245 (PMC4773784; doi:10.3389/fmicb.2016.00245)
Supplement: Supplementary file 1 [file Table_1.DOCX]

Table S1 T90 (expressed in days) and Log_10_ reduction after 63 days of two strains of *L. monocytogenes* in manure and lagoon effluent microcosms at 8 °C and at 20 °C

|  |  |  | Manure-1 | | |  | Manure-2 | | | |  | | Lagoon-1 | | | | | Lagoon-2 | | | |  |
| --- | --- | --- | --- | --- | --- | --- | --- | --- | --- | --- | --- | --- | --- | --- | --- | --- | --- | --- | --- | --- | --- | --- |
| Temperature |  |  | T90 | | Abatement ^c^ | | T90 | | abatement | | | T90 | | | abatement | | T90 | | | abatement | | |
|  | Method | strain | Mean^a^ | (sd)^b^ | mean | (sd) | mean | (sd) | mean | (sd) | | mean | | (sd) | mean | (sd) | mean | | (sd) | mean | (sd) | |
| 8 °C | Culture method | L111r | 22.2 ^d^ | (1.6) | 3.0 | (0.5) | 50.3 ^d^ | (4.9) | 1.3 ^d^ | (0.2) | | 15.4 ^d^ | | (2.3) | 3.9 | (0.7) | 25.8 ^d^ | | (0.5) | 2.7 | (0.1) | |
|  |  | L120r | 20.1 ^d^ | (1.0) | 4.0 | (0.4) | 56.7 ^d^ | (2.1) | 1.1 ^d^ | (0.2) | | 21.4 ^d^ | | (0.6) | 3.3 | (0.1) | 25.5 ^d^ | | (0.6) | 2.2 | (0.3) | |
|  | qPCR | L111r | 22.1 ^d^ | (1.7) | 3.0 | (0.2) ^g^ | >56 ^d^ |  | 0.7 ^d^ | (0.1) | | 19.1 ^d^ | | (0.4) | 2.9 | (0.3) | 37.2 ^d^ | | (3.8) | 1.6 | (0.2) | |
|  |  | L120r | 16.4 ^d^ | (2.3) | 3.8 | (0.2) ^g^ | 56.7^d^ | (2.1) | 1.0 ^d^ | (0.1) | | 15.1 ^d^ | | (1.9) | 2.9 | (0.2) | 37.0 ^d^ | | (2.3) | 1.6 | (0.2) | |
|  | qPCR PMA | L111r | 24.2 ^d^ | (1.7) ^g^ | 3.3 | (0.3) | >63 ^d^ |  | 0.9 ^d^ | (0.2) | | 18.0 ^d^ | | (1.5) ^g^ | 3.2 | (0.3) | 32.0 ^d^ | | (1.5) | 1.9 | (0.1) | |
|  |  | L120r | 13.6 ^d^ | (0.4) ^g^ | 4.4 | (0.5) | 51.7 ^d^ | (5.7) | 1.2 ^d^ | (0.1) | | 12.4 ^d^ | | (1.4) ^g^ | 3.2 | (0.2) | 32.8 ^d^ | | (1.4) | 2.1 | (0.2) | |
|  |  |  |  |  |  |  |  |  |  |  | |  | |  |  |  |  | |  |  |  | |
| 20 °C | Culture method | L111r | 3.7^e^ | (0.9) | 5.4 | (0.2) | 9.2^d^ | (2.1) | 6.5 | (0.3) | | 12.0 ^d^ | | (2.4) | 4.5 | (0.8) | 10.3^d^ | | (0.5) | 5.9 | (0.2) | |
|  |  | L120r | 3.4 ^e^ | (0.4) | 5.6 | (0.3) | 10.5 ^d^ | (0.5) | 5.9 | (0.5) | | 8.5 ^d^ | | (2.2) | 3.5 | (0.2) | 10.8 ^d^ | | (1.5) | 6.0 | (0.6) | |
|  | qPCR | L111r | 2.5 ^e^ | (0.1) | 4.0 | (0.5) | 11.9 ^e^ | (1.2) | 3.9 | (0.2) | | 12.9 ^e^ | | (2.0) | 2.5 | (0.1) | 12.9 ^e^ | | (2.0) | 4.3 | (0.2) | |
|  |  | L120r | 2.4 ^e^ | (0.2) | 4.7 | (0.1) | 12.3 ^e^ | (1.2) | 4.1 | (0.2) | | 8.5 ^e^ | | (1.7) | 2.6 | (0.3) | 11.5 ^d^ | | (1.5) | 4.3 | (0.1) | |
|  | qPCR PMA | L111r | 2.6 ^e^ | (0.2) | >4.6 ^f^ | | 11.6^d^ | (0.6) | 3.8 | (0.1) | | 12.9 ^e^ | | (1.3) ^g^ | 2.8 | (0.3) | 9.2 ^d^ | | (1.2) | 4.4 | (0.4) | |
|  |  | L120r | 2.4 ^e^ | (0.2) | >4.7 ^f^ | | 10.8 ^e^ | (1.5) | 4.1 | (0.1) | | 7.1 ^e^ | | (0.7) ^g^ | 2.8 | (0.3) | 11.0 ^d^ | | (1.3) | 4.3 | (0.2) | |

^a^ Mean of three replicates; ^b^ Standard deviation; ^c^ Log_10_ reduction of cfu or cfu-eq after 63 days of incubation; ^d^ T90 was estimated using the monophasic model;

^e^ T90 was estimated using the biphasic model; ^f^ not detected at day 63 (below the limit of detection: 5 10^3^ cfu-eq mL^-1^), ^g^ mean values between strain L111r and strain L120r differed significantly (p < 0.01, Student's t test)
