# Supplementary material for: Fate of Viable but Non-culturable Listeria monocytogenes in Pig Manure Microcosms
Source: Front Microbiol. 2016 Mar 2;7:245. doi: 10.3389/fmicb.2016.00245 (PMC4773784; doi:10.3389/fmicb.2016.00245)
Supplement: Supplementary file 2 [file Table_2.DOCX]

Table S2 Chemical composition of the two manures and lagoon effluents and concentrations of total bacteria estimated by qPCR_PMA_ at T0 and T63

| Matrix | Temperature  (°C) | time | pH | TS  g/Kg | VS  g/Kg | NTK gN/Kg | TAN g/L | VFA g/L | Total bacteria qPCR_PMA_  (Eq-genome. mL^-1^) |
| --- | --- | --- | --- | --- | --- | --- | --- | --- | --- |
| Manure-1 | 8 | T0 | 7.5 | 15.8 | 8.7 | 1.8 | 1.1 | 0.8 | 6.7 10^9^ |
|  |  | T63 | 7.2 | 11.3 | 5.4 | 2.2 | 0.5 | 0.1 | 2.0 10^9^ |
|  | 20 | T0 | 7.6 | 12.8 | 6.5 | 2.3 | 1.1 | 0.3 | 5.0 10^9^ |
|  |  | T63 | 7.4 | 19.0 | 9.8 | 2.2 | 1.2 | 0.0 | 4.5 10^9^ |
| Manure-2 | 8 | T0 | 7.8 | 26.7 | 14.4 | 3.3 | 2.5 | 0.4 | 5.1 10^9^ |
|  |  | T63 | 7.7 | 22.1 | 12.0 | 3.2 | 2.1 | 0.0 | 4.4 10^9^ |
|  | 20 | T0 | 7.6 | 31.2 | 18.6 | 3.8 | 2.0 | 0.4 | 4.1 10^9^ |
|  |  | T63 | 7.3 | 26.7 | 15.0 | 4.0 | 2.0 | 0.1 | 4.5 10^9^ |
| Lagoon-1 | 8 | T0 | 8.2 | 4.7 | 0.9 | 0.04 | 0.01 | 0.4 | -^*^ |
|  |  | T63 | 8.1 | 4.9 | 0.9 | 0.1 | 0.00 | 0.0 | - |
|  | 20 | T0 | 8.0 | 5.4 | 1.1 | 0.0 | 0.00 | 0.0 | 3.3 10^8^ |
|  |  | T63 | 8.7 | 5.6 | 1.1 | 0.1 | 0.00 | 0.0 | 2.5 10^8^ |
| Lagoon-2 | 8 | T0 | 8.4 | 7.3 | 2.0 | 0.2 | 0.05 | 0.0 | 3.9 10^8^ |
|  |  | T63 | 9.0 | 6.8 | 1.6 | 0.1 | 0.05 | 0.0 | 3.9 10^8^ |
|  | 20 | T0 | 8.4 | 6.7 | 1.6 | 0.1 | 0.08 | 0.0 | 4.9 10^8^ |
|  |  | T63 | 9.0 | 6.8 | 1.6 | 0.1 | 0.00 | 0.0 | 4.5 10^8^ |

* no data
