# Supplementary material for: Fate of Viable but Non-culturable Listeria monocytogenes in Pig Manure Microcosms
Source: Front Microbiol. 2016 Mar 2;7:245. doi: 10.3389/fmicb.2016.00245 (PMC4773784; doi:10.3389/fmicb.2016.00245)
Supplement: Supplementary file 3 [file Table_3.DOCX]

Table S3 Phylogenetic composition of the bacterial communities in the manure and lagoon effluent microcosms inoculated with strain L111r

|  | Manure-1 | | | | Manure-2 | | | | Lagoon-1 | | | | Lagoon-2 | | | |
| --- | --- | --- | --- | --- | --- | --- | --- | --- | --- | --- | --- | --- | --- | --- | --- | --- |
| Phyla | 8 °C | | 20 °C | | 8 °C | | 20 °C | | 8 °C | | 20 °C | | 8 °C | | 20 °C | |
|  | T0 | T63 | T0 | T63 | T0 | T63 | T0 | T63 | T0 | T63 | T0 | T63 | T0 | T63 | T0 | T63 |
| *Acidobacteria* | 0.02 | 0.04 | 0.07 | 0.04 | 0.01 | 0.00 | 0.00 | 0.00 | 0.02 | 0.18 | 0.00 | 0.00 | 0.05 | 0.07 | 0.01 | 0.01 |
| *Actinobacteria* | 5.52 | 6.44 | 4.43 | 4.89 | 1.39 | 2.07 | 1.50 | 1.14 | 5.19 | 16.33 | 0.52 | 0.65 | 2.69 | 2.77 | 0.40 | 0.61 |
| *Bacteroidetes* | 15.99 | 17.34 | 20.24 | 17.23 | 22.59 | 19.26 | 21.78 | 22.96 | 7.05 | 20.29 | 6.19 | 9.47 | 13.90 | 18.57 | 1.78 | 22.04 |
| *Chlorobi* | 0.15 | 0.26 | 0.18 | 0.19 | 0.07 | 0.05 | 0.05 | 0.10 | 0.13 | 0.05 | 0.01 | 0.03 | 0.12 | 0.07 | 0.02 | 0.05 |
| *Chloroflexi* | 0.05 | 0.06 | 0.09 | 0.14 | 0.15 | 0.05 | 0.13 | 0.11 | 0.01 | 0.01 | 0.00 | 0.00 | 0.13 | 0.08 | 0.00 | 0.00 |
| *Cyanobacteria* | 0.00 | 0.00 | 0.00 | 0.00 | 0.00 | 0.00 | 0.00 | 0.00 | 2.94 | 0.01 | 0.03 | 0.00 | 0.01 | 0.01 | 0.01 | 0.00 |
| *Fibrobacteres* | 0.31 | 0.75 | 0.48 | 0.34 | 0.32 | 0.18 | 0.52 | 0.67 | 0.01 | 0.35 | 0.00 | 0.00 | 0.35 | 0.26 | 0.04 | 0.01 |
| *Firmicutes* | 39.57 | 48.90 | 43.41 | 49.95 | 56.84 | 66.07 | 57.29 | 54.81 | 72.50 | 23.35 | 10.68 | 24.96 | 45.48 | 32.23 | 10.04 | 14.09 |
| *Fusobacteria* | 0.04 | 0.03 | 0.23 | 0.02 | 0.02 | 0.04 | 0.04 | 0.05 | 0.03 | 0.01 | 0.02 | 0.02 | 0.10 | 0.13 | 0.01 | 0.01 |
| GN02 | 0.01 | 0.01 | 0.01 | 0.02 | 0.02 | 0.02 | 0.01 | 0.01 | 0.10 | 0.05 | 0.00 | 0.00 | 0.10 | 0.06 | 0.01 | 0.00 |
| GN04 | 0.08 | 0.15 | 0.08 | 0.13 | 0.00 | 0.00 | 0.00 | 0.00 | 0.09 | 0.09 | 0.01 | 0.00 | 0.00 | 0.00 | 0.00 | 0.00 |
| *Gemmatimonadetes* | 0.11 | 0.15 | 0.14 | 0.21 | 0.01 | 0.01 | 0.00 | 0.01 | 0.12 | 0.95 | 0.02 | 0.02 | 0.19 | 0.36 | 0.03 | 0.07 |
| *Lentisphaerae* | 0.14 | 0.12 | 0.24 | 0.29 | 0.07 | 0.13 | 0.10 | 0.07 | 0.00 | 0.00 | 0.00 | 0.00 | 0.00 | 0.00 | 0.00 | 0.00 |
| *Nitrospirae* | 0.08 | 0.12 | 0.13 | 0.16 | 0.00 | 0.00 | 0.00 | 0.00 | 0.01 | 0.01 | 0.00 | 0.00 | 0.00 | 0.00 | 0.00 | 0.00 |
| *Proteobacteria* | 25.94 | 12.34 | 13.86 | 11.98 | 6.40 | 5.05 | 6.33 | 6.89 | 10.51 | 35.81 | 79.69 | 60.07 | 31.18 | 40.01 | 83.00 | 54.09 |
| SAR406 | 0.09 | 0.20 | 0.15 | 0.05 | 0.11 | 0.02 | 0.07 | 0.09 | 0.00 | 0.00 | 0.00 | 0.00 | 0.00 | 0.00 | 0.00 | 0.00 |
| SR1 | 0.03 | 0.02 | 0.05 | 0.01 | 0.05 | 0.03 | 0.05 | 0.05 | 0.00 | 0.00 | 0.00 | 0.00 | 0.01 | 0.01 | 0.00 | 0.00 |
| *Spirochaetes* | 0.83 | 1.50 | 1.43 | 0.61 | 0.64 | 0.34 | 0.70 | 1.08 | 0.02 | 0.07 | 0.01 | 0.04 | 0.21 | 0.17 | 0.01 | 0.24 |
| TM6 | 0.00 | 0.00 | 0.01 | 0.01 | 0.01 | 0.01 | 0.00 | 0.00 | 0.15 | 0.23 | 0.01 | 0.00 | 0.06 | 0.06 | 0.00 | 0.00 |
| TM7 | 0.02 | 0.02 | 0.02 | 0.04 | 0.00 | 0.00 | 0.00 | 0.00 | 0.35 | 0.84 | 0.01 | 0.00 | 0.04 | 0.02 | 0.01 | 0.00 |
| Tenericutes | 2.34 | 2.17 | 4.22 | 1.76 | 2.87 | 0.33 | 2.47 | 2.68 | 0.23 | 0.59 | 1.02 | 2.01 | 1.25 | 1.54 | 2.99 | 3.30 |
| WWE1 | 2.25 | 3.78 | 3.92 | 4.63 | 3.35 | 2.89 | 3.84 | 4.42 | 0.02 | 0.02 | 0.01 | 0.05 | 0.25 | 0.10 | 0.03 | 0.55 |
| Others | 6.41 | 5.60 | 6.63 | 7.32 | 5.03 | 3.47 | 5.10 | 4.85 | 0.53 | 0.76 | 1.77 | 2.66 | 3.88 | 3.47 | 1.61 | 4.93 |
